# P-1616. The Impact of SARS-COV-2 Pre-admission Screening on Hospital-Onset SARS-COV-2 Infections at an NYC Health System

**DOI:** 10.1093/ofid/ofaf695.1793

**Published:** 2026-01-11

**Authors:** Jordan A Ehni, Bernard Camins, Ankit Sakhuja, Nimay Hizare

**Affiliations:** Mount Sinai Health System - Mount Sinai Downtown, Brooklyn, NY; Icahn School of Medicine at Mount Sinai, New York, NY; Icahn School of Medicine at Mount Sinai, New York, NY; Icahn School of Medicine at Mount Sinai New York, New York, JERSEY CITY, New Jersey

## Abstract

**Background:**

Since the COVID-19 pandemic began hospitals have implemented various infection prevention practices to prevent its spread. Among these practices include SARS-COV-2 testing of all patients before hospital admission. The impact of pre-admission screening on preventing SARS-COV-2 nosocomial transmission remains unclear, and if the possible impact outweighs the financial, labor, and patient throughput costs. The purpose of this study was to analyze the impact that pre-admission SARS-COV-2 screening had on hospital-onset (HO) COVID-19 rates.

**Figure:**
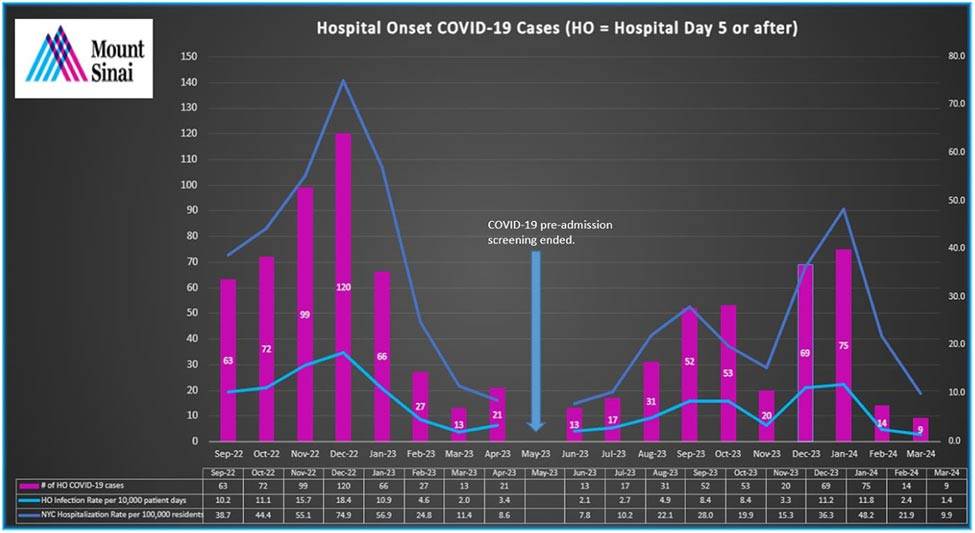

**Methods:**

A retrospective data analysis was performed of patients admitted to a New York City (NYC) hospital system from 9/1/2022 through 3/31/2024 using an infection prevention surveillance system. From 9/1/2022 through 4/30/2023 all patients were tested for SARS-COV-2 before admission. In May 2023, pre-admission COVID-19 screening was phased out. Infections were classified as HO if collected on or after hospital day 5 with no previous positive test thirty days prior. Spearman’s Rank Correlation Coefficient was used to determine the relationship between the study variables.

**Results:**

A comparison of the pre-admission testing period to when it ceased showed a 32.95% reduction occurred in the HO COVID-19 rate (12.29 to 8.24 cases per 10,000 patient days) while a larger 42.28% reduction occurred in the NYC COVID-19 hospitalization rate (30.43 to 17.62 hospitalizations per 100,000 residents). The relative ratio of the HO COVID-19 rate over the NYC hospitalization rate increased slightly from 0.40 to 0.47. A strong positive correlation of r = 0.89 (p-value < 0.0001) was found between the HO COVID-19 rate and the NYC COVID-19 hospitalization rate.

**Conclusion:**

The results indicated that HO COVID-19 rates are strongly driven by community COVID-19 rates and that pre-admission testing was shown to have a slight impact on preventing HO COVID-19. Hospital visitor restrictions, universal masking, and other prevention practices focusing on community source control should be considered, especially when COVID-19 community rates are elevated.

**Disclosures:**

All Authors: No reported disclosures

